# Vaccine Hesitancy in Central Switzerland: Identifying and Characterizing Undervaccinated Children in a Pediatric Emergency Department

**DOI:** 10.3390/pediatric15040064

**Published:** 2023-12-05

**Authors:** Anika Ménétrey, Markus A. Landolt, Michael Buettcher, Thomas J. Neuhaus, Leopold Simma

**Affiliations:** 1 Department of Pediatrics, Children’s Hospital Lucerne, Spitalstrasse, 6000 Lucerne, Switzerland; 2 Department of Neurology, University Children’s Hospital Zurich, 8032 Zurich, Switzerland; 3 Department of Psychosomatics and Psychiatry, University Children’s Hospital Zurich, 8032 Zurich, Switzerland; 4 Division of Child and Adolescent Health Psychology, Department of Psychology, University of Zurich, 8032 Zurich, Switzerland; 5 Children’s Research Center, University Children’s Hospital of Zurich, University of Zurich, 8032 Zurich, Switzerland; 6 Pediatric Infectious Diseases Unit, Children’s Hospital Lucerne, Cantonal Hospital Lucerne, 6000 Lucerne, Switzerland; michael.buettcher@luks.ch; 7 Department of Pediatric Pharmacology and Pharmacometrics, University Children’s Hospital Basel, University of Basel, 4056 Basel, Switzerland; 8 Emergency Department, University Children’s Hospital Zurich, Steinwiesstrasse 75, 8032 Zurich, Switzerland

**Keywords:** vaccination, child, pediatric emergency department, vaccine hesitancy, pediatrics

## Abstract

Vaccinations play an important role in the prevention of potentially fatal diseases. Vaccine hesitancy has become an important problem both in the public discourse and for public health. We aimed to identify and characterize this potentially unvaccinated or incompletely vaccinated group of children presenting to the pediatric emergency department (PED) of the tertiary children’s hospital in central Switzerland, a region that has anecdotally been claimed as a hotspot for vaccine hesitancy. All patients presenting to the PED (N = 20,247) between September 2018 and September 2019 were screened for their vaccination status and categorized as incomplete, unvaccinated, or fully vaccinated in a retrospective cohort study. Some 2.6% (n = 526) visits to the PED were not or incompletely vaccinated according to age, or their vaccination status was unknown. Most of the children in the cohort were not critically ill, and the minority had to be hospitalized. Undervaccinated patients were overrepresented in rural areas. Of all cohort visits, 18 (3.4%) patients received opportunistic vaccination in the PED. No cases of vaccine-preventable diseases were observed. In summary, incompletely vaccinated and unvaccinated status was less frequent than initially expected. The PED may play a role in increasing vaccination coverage by providing opportunistic vaccinations.

## 1. Introduction

Vaccinations play a key role in the prevention of potentially life-threatening infectious diseases. Preventing potentially fatal infectious diseases has been a major concern of humankind for centuries, and vaccines have proven to be the most cost-effective method [1]. Initially known as “inoculation” fighting smallpox, the general principle of creating immunity by exposure was understood in the 18th century. However, concerns about vaccines have existed since their first use [2]. Prior to the SARS-CoV-2 pandemic, pediatric and public health research reported an increase in number of vaccine-skeptical/hesitant parents in recent decades [3,4,5,6,7]. Certain infectious diseases, such as measles, were close to eradication thanks to vaccination. Their re-emergence is likely due to lower public awareness of this potentially fatal infectious disease and consequent lower vaccination rates [8]. In 2019, the WHO reported that 14 million children had not received an initial dose of the diphtheria-tetanus-polio (DTP) vaccine due to lack of access to immunization or health services and an additional 5.7 million children worldwide were only partially vaccinated [9]. In addition, high-income countries exhibit an increasing trend towards partially vaccinated or unvaccinated children due to parents’ or caregivers’ decisions, despite access to modern medical facilities [10].

During the SARS-CoV-2 pandemic, public debate transformed vaccinations into a mainstream topic of conversation [11] and vaccine hesitancy and vaccination skepticism have moved to center stage. Anecdotally, the catchment area of the Children’s Hospital Lucerne, which contained 866,596 inhabitants on 31 December 2020, has a high rate of unvaccinated or partially vaccinated children [12].

The occasional emergence of vaccine-preventable diseases (VPDs) may have shaped this reputation, with cases of tetanus at our hospital and the 2006–2009 measles epidemic in central Switzerland [13]. For most VPDs, with a vaccination coverage of 95%, so-called herd protection is achieved. The Swiss Federal Office of Public Health (FOPH) reports regularly on vaccination coverage. The report for 2019 shows a vaccination coverage of around 90%, depending on the targeted disease, in the catchment area of the Children’s Hospital in Lucerne. Recommended basic vaccinations in 2 year olds range from 92–96% for basic vaccination; supplementary immunizations such as pneumococcal vaccine are reported to be as low as 68% [14].

So far, few publications have reviewed vaccination status in pediatric emergency department (PED) populations [15,16]. To our knowledge, there are no data available so far on the characteristics of a distinct population of children of vaccine-hesitant parents.

Our objective was to analyze and characterize the features of incompletely or unvaccinated children treated in the only tertiary PED in central Switzerland and where possible to identify vaccine-hesitant regional clusters.

## 2. Materials and Methods

### 2.1. Study Design

We conducted a retrospective cohort study of data collected during triage in the PED between 19 September 2018 and 21 September 2019. Informed consent for use of anonymized patient data was waived by the ethics committee. This study was approved by the ethics committee responsible for central Switzerland (EKNZ 2019-01875). This article was written in accordance with the STROBE guidelines [17].

### 2.2. Setting

The PED has an annual census of 21,000 patients. It serves an urban and rural population in a geographically confined area and treats both patients under 16 years and older patients with chronic pediatric conditions.

All patients are seen first by administrative staff and the triage nurse. During the period of this study, global triage assessment regularly involved collecting the following data on paper-based triage notes: reason for consultation, global triage assessment (Australasian triage scale—ATS: highest acuity = triage 1, lowest acuity = triage 5) [18], past medical history, and short medical record of events and procedures in the PED. For administrative purposes, patients were traditionally grouped into medical and surgical presentations.

### 2.3. Swiss Vaccination Recommendations

Swiss vaccination recommendations are categorized as basic and recommended supplementary vaccinations. During the study period, diphtheria, tetanus, acellular pertussis, hemophilus influenzae type b, and poliomyelitis combination vaccine (DTaP-HiB-IPV) doses were recommended at 2, 4, 6, and 15–24 months of age, DTaP at 4–6 years and 11–15 years, and measles, mumps, and rubella vaccine (MMR) doses at 12 and 15–24 months. Recommended supplementary vaccinations included pneumococcus (PCV) at 2, 4, and 12 months, and meningococcal (MCV) at 24 months. Vaccination against rotavirus is not part of the national schedule. The varicella vaccine is recommended only for teenagers without documented prior infection [19]. Only vaccinations in the recommended schedule were considered, as only these could be recorded reliably during the triage process.

During the study period, VPDs were defined as infections with diphtheria, tetanus, pertussis, hemophilus influenzae type b, poliomyelitis, measles, mumps, rubella, pneumococcus, and meningococcus.

### 2.4. Inclusion Criteria and Data Collection

All patients presenting to the PED were routinely screened for their vaccination status by the triage nurse. Patients who presented to the PED several times were analyzed each visit. Triage forms were completed as described above. All triage forms were screened, and data were included in the analysis if patients were unvaccinated, incompletely vaccinated, or had a void vaccination status. Children under the age of 2 months were excluded, as the Swiss national authority of Public Health (BAG) does not recommend any vaccinations under this age [19]. Void vaccination status was double checked with physicians’ notes in the electronic medical record (EMR).

For our study on vaccine hesitancy, we used all paper-based triage forms for the retrospective analysis. Patient data of patients discharged from the PED (outpatients) were available for analysis over a 12-month period; data from admitted PED patients (inpatients) was available from February 2019 to September 2019 (7 months).

Because our study sought to reflect real-world conditions, our study relied solely on information provided during the patient’s visit. This information was usually supplied verbally by caregivers, and vaccination certificates were rarely available in PED visits.

As routine in the busy pediatric emergency medicine practice, we relied mainly on information given verbally by the caregiver. Some parents may have produced a vaccination certificate during the visit. In the absence of a national childhood vaccination register, we must emphasize that we were unable to externally verify the vaccination data. As this was a retrospective chart-based study, our research did not involve direct contact to all private physicians and pediatricians of all patients in the cohort.

### 2.5. Variables

All triage notes were screened for missing or negative comments on vaccination status. We collected the following variables: age, disposition, sex, time and date of presentation, triage category by ATS, vaccination status, treatment, presence of chronic disease, and being a frequent PED patient. Frequent use was defined as three or more visits per year.

**Figure 1:**
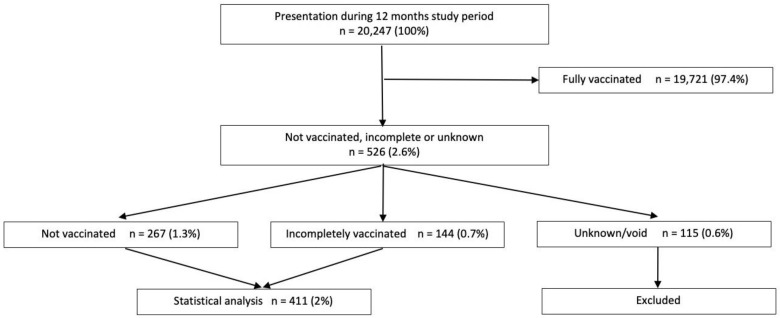
Patient flow chart.

The vaccination status of the incompletely vaccinated or unvaccinated patients was subcategorized in five groups according to triage notes: no vaccinations, tetanus vaccination only, incomplete, or unspecified vaccination status, vaccination status without MMR, or undocumented status. For statistical analysis, these groups were merged into three categories: no vaccinations, incomplete, and unknown or undocumented (Figure 1). Children with unknown vaccination status were excluded from statistical analysis.

Our geographical analysis used data for a communal aggregation level from the Swiss Federal Statistical Office (FSO) [20]. The dataset published in 2012 divides the Swiss communes into three categories: urban, intermediate, and rural. This categorization is derived from a subdivided classification in which the communes are classified depending on population density, total population, and accessibility criteria.

### 2.6. Statistical Methods

Paper-based information was entered in a Microsoft Excel spreadsheet (Excel 2019, Version 16.0, Microsoft, Redmond, WA, USA). Further patient details were extracted from the EMR. After completion the data were extracted and the dataset was anonymized.

Unknown vaccination status was handled as missing information and was excluded from further statistical analysis. Due to the nature of the data, missing values could not be imputed.

Data were analyzed using the IBM SPSS Statistical Software Package for Macintosh (version 26). Analyses were performed with two-sided tests and a *p* < 0.05 was considered significant for all tests. Groups were compared using chi-square tests for categorical variables and Student *t* tests or ANOVAs for continuous variables.

Post codes of patients were allocated to urbanization classification [21] according to the DEGURBA typology by Eurostat. The Swiss data in the STATPOP 2011 database from the Swiss FSO was used to classify urban, intermediate, and rural Swiss postcodes. We analyzed presentations by postcode with SPSS and generated a map with free and open source QGIS Version 3.20 [22], with the postcode data from the Swiss cadastral system [23].

## 3. Results

### 3.1. Participants

During the 12-month study period, a total of 20,247 patient visits were recorded. Of all visits, 526 (2.6%) visits fulfilled the inclusion criteria of incomplete or no vaccinations. Of this subgroup, 411 visits (78.1% [cohort]) had either incomplete (27.4%) or no vaccination (50.7%). For 115 visits (21.9% [cohort]; 0.6% [all visits]), the vaccination status notes were void, resulting in an unknown vaccination status. (Figure 1).

### 3.2. Descriptive Data

The main patient characteristics of the sample are shown in Table 1.

**Figure 2:**
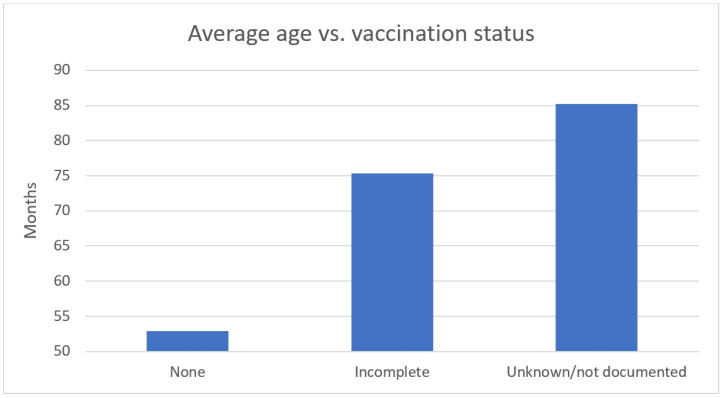
Average age vs. vaccination status (age in months).

In our sample, slightly fewer children were female (44% among the 411 visits analyzed) among the incompletely vaccinated and unvaccinated. The average age of the undervaccinated subgroup was 66 months (5.5 years of age) (Figure 2).

Males were more often incompletely vaccinated or unvaccinated than females, but this trend was not statistically significant (Χ^2^ (2, n = 411) = 0.185, *p* = 0.667 (*p* value 0.076). A minority of chronically ill patients were unvaccinated, but the number was not statistically significant (Χ^2^ (2, n = 411) = 0.009, *p* = 0.925 (*p* value 0.246).

Visits in the sample rarely occurred during the night (11.4%) and were evenly distributed over the whole week. The day of presentation showed no statistically significant changes and no preferred day for presentation of unvaccinated children.

**Figure 3:**
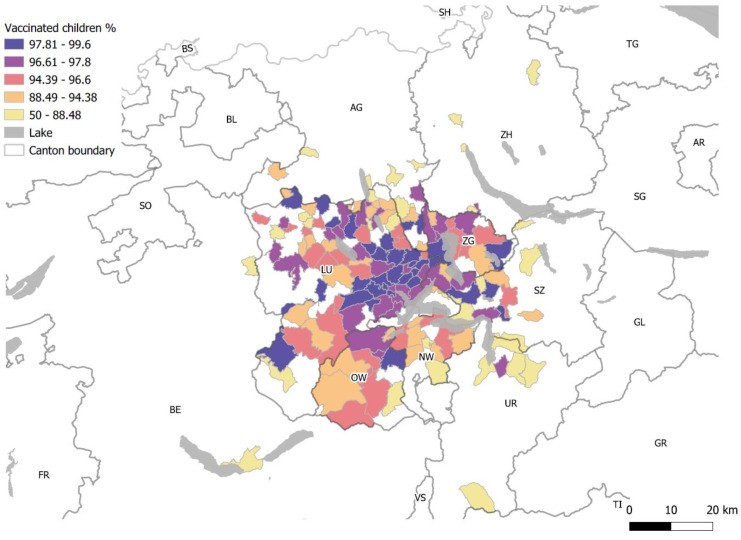
Map of central Switzerland: vaccination rate (%) of the PED visits by post codes. Cantons: AG: Aargau; BE: Berne; LU: Lucerne; NW: Nidwalden; OW: Obwalden; SZ: Schwyz; UR: Uri; ZG: Zug; ZH: Zurich.

Analysis of geographical distribution demonstrates that patient groups from urban areas tend to be more thoroughly vaccinated than those living in rural areas. (Table 2 and Figure 3). Patient groups from rural postcodes had a significantly higher proportion of incompletely vaccinated and unvaccinated children (*p* = 0.0012).

More than two thirds were low-acuity presentation visits. None of the unvaccinated children were triaged as high-acuity presentations. However, high-acuity presentations often lacked documentation of vaccination status.

Some 373 (70.9% [cohort], 1.8% [all visits]) were outpatients visits, and 153 (29.1% [cohort], 0.8% [all visits]) were inpatient admissions. Overall, a greater proportion of outpatient visits were unvaccinated (56.6% [cohort], 1% [all visits]) than among the inpatient cohort (36.6% [cohort], 0.3% [all visits]). The proportion of whose vaccination status was unknown was higher in the inpatient group. This difference was statistically significant (Χ^2^ (2, n = 526) = 41.684, *p* < 0.001). The majority (53%) of undocumented vaccination status were hospital admissions.

**Figure 4:**
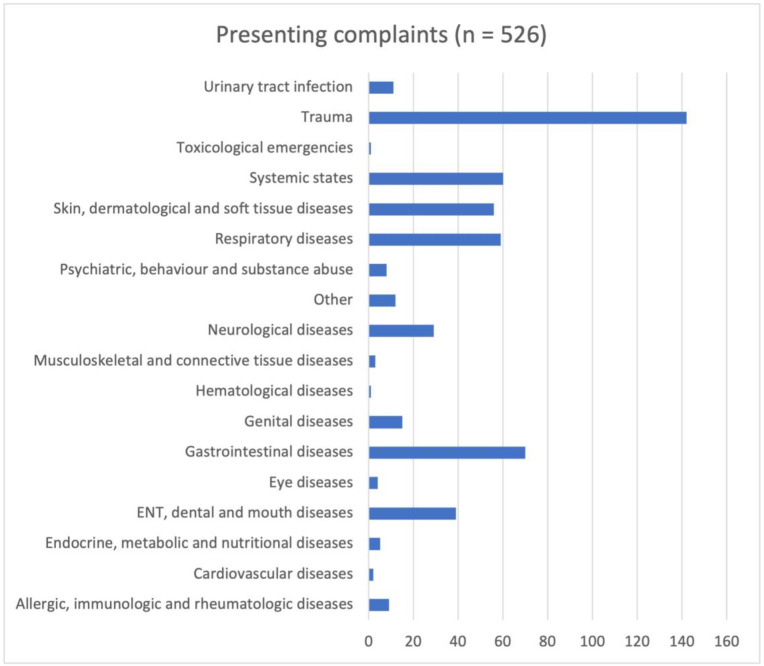
Cohort grouped in Pediatric Emergency Care Applied Research Network (PECARN) diagnosis categories (absolute numbers).

No difference in vaccination status was found for medical or surgical presentation groups (Χ^2^ (2, n = 411) = 0.475, *p* = 0.491 (*p* value = 0.065). Allocation into Pediatric Emergency Care Applied Research Network (PECARN) diagnosis categories [24] showed most visits occurred for trauma, followed by gastrointestinal problems, systemic states such as fever, viral, bacterial, or fungal infection or respiratory diseases (Figure 4). In our cohort, there were no cases of VPD.

**Figure 5:**
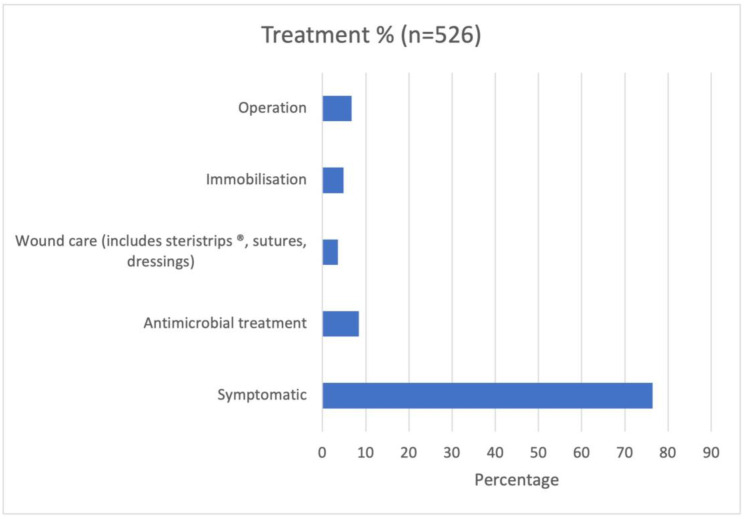
Treatment modality (N = 526, percentage).

The most frequent treatments were symptomatic treatment only (76.4%), antimicrobial treatment (8.4%), operation (6.7%), immobilization (4.9%), and wound care (including Steristrips^®^, sutures, dressings) (3.6%) (Figure 5).

The above-mentioned treatments were more likely in unvaccinated children (overall 65%), except for operative treatment, which occurred in equal proportions in incompletely vaccinated and unvaccinated groups. Outpatient and inpatient visits had statistically significant differences in treatment (Χ^2^ (6, n = 411) = 4.004, *p* = 0.676 (*p* value = 0.017)), with operative treatment being the most frequent treatment after symptomatic treatment in inpatients.

Of our cohort, 18 (3.4% [cohort], 0.1% [all visits]) patients were vaccinated during their visit. All vaccinations were administered in the PED for lacerations due to lacking or incomplete tetanus protection.

Subgroup analysis of incomplete vaccination versus no vaccination showed no statistical difference of the following variables: sex (*p* = 0.373), triage category (*p* = 0.529), pediatric vs. surgical (*p* = 0.279), disposition (*p* = 0.208), day of presentation (*p* = 0.565), and treatment of the subgroups (*p* = 0.676).

## 4. Discussion

### 4.1. Key Results

Only 2.5% of all visits to the PED were incompletely vaccinated or unvaccinated (undervaccinated subgroup). Within this undervaccinated subgroup, the majority of children were unvaccinated (50%), followed by a group of incomplete vaccination status, which also encompasses all the patients who did not adhere to vaccination schedules.

We found no cases of VPD in the period observed, and most presentations were low acuity. Undervaccinated children had an average age of 66 months (5.5 years of age) (Figure 2).

A Swiss study that used insurance data from 2010 reported that only 40.9% of children were up to date with their vaccination status at the age of 25 months [25]. In a study covering 2010 to 2017 also analyzing insurance data of a Swiss cohort, only seven out of 10 children had an up-to-date vaccination status at the age of 37 months [26].

In our sample, most of the incompletely vaccinated children lacked MMR, a phenomenon that was also reported in Swiss studies in 2016 and 2020, which identified the problem of postponing or omitting MMR vaccines [26,27]. Outbreaks of VPDs during the last few years in high-income countries could be associated with very low vaccination uptake in the affected communities [28,29,30,31,32].

We observed an overrepresentation of undervaccinated children in rural areas, taking into consideration that residents of these areas tend to use the PED less frequently. However, with an annual patient volume of 21,000 patients in the PED and a vaccination rate of 92%, theoretically, a number of around 1600 patients with partial or no vaccination protection could be assumed. The actual number in our data is much lower; however, a PED population does not permit extrapolation to the general population. Regional differences in vaccination status were also noticed by different studies in Switzerland [14,26]. Schneider et al. showed that around 5% of their cohort living in central Switzerland were completely unvaccinated by the age of 37 months [26]. Regional differences could be related to the factors convenience and complacency as elaborated in the 3Cs model of vaccination hesitancy by the SAGE Working Group on Vaccine Hesitancy [33]. However, hesitancy is a continuum and it is critical to differentiate hesitancy from other reasons for undervaccination [33]. Thus, undervaccination should not be equated with vaccine hesitancy, as it may be influenced by various factors, including access issues and medical contraindications.

Intuitively, one may speculate that unvaccinated children present with VPDs and tend to be more severely ill. This was not observed in our sample. However, we found that undocumented vaccination status was more frequent in children with severe illness or injury, suggesting that the important task of documenting a vaccination status is not the priority in life-threatening situations. The percentage of high-acuity cases was twice that of a cohort of critically ill children in our ED [34]. However, the absolute number of five visits in the highest acuity category was still very low.

Apart from high acuity, age may play a role in the documentation of vaccination status, as shown in Figure 2. Possible reasons may be that parents may not remember details about vaccinations or that vaccination status may be lower on the triage nurse’s priority list with older children and adolescents.

In our cohort, the most common presenting complaint was injury, a leading cause of presentation in most PEDs [35]. There was also an even distribution of presentations over the week, even on Thursdays, when many clinics of pediatricians and family doctors in Switzerland traditionally close in the afternoon.

In our cohort, 18 patients (0.1%) of all cohort visits received opportunistic vaccination in the PED due to lacking tetanus cover. Acceptance of these unplanned vaccinations by both parents and staff during hospital stay has been demonstrated in studies. Parents of chronically ill patients would particularly benefit from opportunistic vaccinations during hospital stays [36]. To tackle this, a quality improvement program could be piloted where parents are provided with informational material (e.g., leaflet on the misconceptions around MMR and autism, etc.) and offered the opportunity to catch up on missed vaccinations. If successful, such a program could be extended to other hospitals. Similar programs are already established in the United States for influenza vaccinations [37]. Nevertheless, both acute illness and focus on acute rather than preventive care has been identified as a major challenge to vaccination in EDs [38].

### 4.2. Limitations

Our study has several limitations. We examined a small population of a defined area in central Switzerland which enabled us to link rates of vaccination areas of residence. However, the PED population is not a representative sample of the general population and thus results are not generalizable. Thus, this study does not permit extrapolation on vaccination coverage of the pediatric population in our catchment area. A major limitation was incomplete documentation; the vaccination status was not correctly documented for 20% in our cohort. In the inpatient group, information about vaccination status was lacking in as many as 40% of those cases.

We also relied on the vaccination status reported by caregivers and were unable to verify due to the retrospective study design. Due to the absence of a national vaccination registry, we could not verify the vaccination status independently, which may introduce uncertainties. The lack of verification precludes assessment for potential conformity bias.

Nurses, medical students, and medical staff were not briefed prior to data collection. This fact retrospectively contributes to an observation bias. Although this reflects real-life conditions in a busy PED, for future research, a prospective design or a quality improvement project would likely result in better documentation.

The numbers of outpatients and inpatients are not directly comparable, as data for inpatients was not available for the whole study period.

Due to limited statistical power (attributable to the small size of the unvaccinated group) we might have not detected certain significant and, therefore, meaningful differences between the groups.

Our finding that children living in urban areas tend to be more thoroughly vaccinated than in more rural areas (Table 2) may be influenced by a bias due to differences in the frequency of presentations to the PED. Furthermore, additional sociodemographic data was unavailable because it is not routinely collected. Likewise, our retrospective dataset lacks information about the factors why children in our cohort did not receive vaccinations.

Finally, in the absence of a comprehensive electronic medical record, we were unable to retrospectively compare our cohort to the overall consultations in the PED as studies such as Smith et al.’s did [5]. It is also unclear whether our cohort is representative of the overall population of central Switzerland.

Despite these limitations, our study contributes valuable insights into vaccine hesitancy in a visit to a PED in Central Switzerland, highlighting the challenges of real-world data collection in the absence of a comprehensive vaccination registry.

### 4.3. Interpretation

Incomplete or lacking vaccination status was by far less common than both anecdotal evidence and extrapolation of vaccine coverage suggested. The reasons underlying in-complete or absent vaccination remain diverse. In a systematic review in 2018, Larson et al. described trust as being one of the key factors in parental decision making about vaccination [39]. Trusting the vaccine itself, the provider, and the information given to the patients and their caregivers can make all the difference in the decision making whether to vaccinate a child [40].

Nevertheless, vaccine hesitancy remains a daily issue for medical professionals [10,41,42]. Medical professionals remain key players in informing patients and others about vaccine safety and efficacity [40]. Constant efforts are required by PEM providers to take opportunities to educate parents and caretakers about risks of VPD and vaccine side effects [43,44].

## 5. Conclusions

Lack of vaccination was less frequent than expected in a population of patients in a PED in central Switzerland, but it remains an issue of concern in a high-income country. Our data demonstrates that a national vaccination registry would help to gain more insight into actual vaccination rates and would enable further in-depth research. Education about vaccines and their safety and risk–benefit evaluation needs to be kept in mind in daily medical practice. Even a short stay in the PED or a longer stay at the hospital may offer an opportunity to approach vaccine-hesitant caregivers.

## Figures and Tables

**Table 1:** Patient characteristics: Total and undervaccinated.

Age: Average (months) 66 [CI (95% 61;71)]; SD 55			
	n	%	
Total visits (N = 20,247 = all visits)			
Completely vaccinated	19,721	97.4	
No vaccination or incomplete	526	2.6	
Vaccination status (N = 526 = cohort)		% all visits	% cohort
None	267	1.3	50.8
Incomplete	144	0.7	27.3
Unknown/not documented	115	0.6	21.9
Sex			
Male	289	1.4	54.9
Female	237	1.2	45.1
Disposition			
Discharged from PED	373	1.8	70.9
Admission	153	0.8	29.1
Time of presentation			
Morning shift (8–16)	220	1.1	41.8
Evening shift (16–23)	246	1.2	46.8
Night shift (23–8)	60	0.3	11.4
Triage category			
1 (highest acuity)	5	0.02	1
2	45	0.2	8.6
3	97	0.5	18.4
4	182	0.9	34.6
5 (lowest acuity)	189	0.9	35.9
Unknown/not classified	8	0.04	1.5
Subspecialty			
Pediatric	285	1.4	54.2
Pediatric Surgery	241	1.2	45.8
Chronic disease			
Yes	25	0.1	4.8
No	501	2.5	95.2
Day			
Monday	66	0.3	12.5
Tuesday	76	0.4	14.4
Wednesday	83	0.4	15.8
Thursday	75	0.4	14.3
Friday	62	0.3	11.8
Saturday	85	0.4	16.2
Sunday	79	0.4	15

**Table 2:** Geographical distribution by degree of urbanization.

	Urban	Suburban	Rural	Total	
Vaccinated	7974	7905	3778	19,657	*p* = 0.00012
Unvaccinated or incomplete	137	160	111	408	
Total	8111	8065	3889	20,065 *	

* Patients residing outside Switzerland were excluded.

## Data Availability

The datasets generated an/or analyzed during the current study are not publicly available as this is beyond the scope of the ethics approval. The corresponding author may be contacted to provide anonymized data upon reasonable request.

## References

[1] Maciosek M.V., Edwards N.M., Coffield A.B., Flottemesch T.J., Nelson W.W., Goodman M.J., Solberg L.I. (2006). Priorities among effective clinical preventive services: Methods. Am. J. Prev. Med..

[2] Flemming A. The Origins of Vaccination. Nature Portfolio 2020. https://www.nature.com/immersive/d42859-020-00005-8/index.html.

[3] Fournet N., Mollema L., Ruijs W.L., Harmsen I.A., Keck F., Durand J.Y., Cunha M.P., Wamsiedel M., Reis R., French J. (2018). Under-vaccinated groups in Europe and their beliefs, attitudes and reasons for non-vaccination; two systematic reviews. BMC Public Health.

[4] Sandhofer M.J., Robak O., Frank H., Kulnig J. (2017). Vaccine hesitancy in Austria: A cross-sectional survey. Wien. Klin. Wochenschr..

[5] Smith P.J., Chu S.Y., Barker L.E. (2004). Children who have received no vaccines: Who are they and where do they live?. Pediatrics.

[6] Siddiqui M., Salmon D.A., Omer S.B. (2013). Epidemiology of vaccine hesitancy in the United States. Hum. Vaccin. Immunother..

[7] Luthy K.E., Beckstrand R.L., Peterson N.E. (2009). Parental Hesitation as a Factor in Delayed Childhood Immunization. J. Pediatr. Health Care.

[8] Posfay-Barbe K.M. (2012). Infections in pediatrics: Old and new diseases. Swiss. Med. Wkly.

[9] World Health Organisation (2022). Immunization Coverage. https://www.who.int/news-room/fact-sheets/detail/immunization-coverage.

[10] Di Pietro M.L., Poscia A., Teleman A.A., Maged D., Ricciardi W. (2017). Vaccine hesitancy: Parental, professional and public responsibility. Ann. Ist. Super Sanita.

[11] Troiano G., Nardi A. (2021). Vaccine hesitancy in the era of COVID-19. Public Health.

[12] Swiss Federal Office for Statistics (2020). Balance of the Permanent Resident Population by Canton. https://www.bfs.admin.ch/asset/de/23064904.

[13] Donas A., Marty-Nussbaumer A., Roost H.P., Neuhaus T.J. (2014). Measles epidemic in a highly developed country: Low mortality, high morbidity and extensive costs. Klin. Padiatr..

[14] Swiss Federal Office of Public Health Table of Vaccination Coverage of 2-, 8- and 16-Year-Old Children in Switzerland, 1999–2021. https://www.bag.admin.ch/bag/de/home/gesund-leben/gesundheitsfoerderung-und-praevention/impfungen-prophylaxe/informationen-fachleute-gesundheitspersonal/durchimpfung.html.

[15] Cove L.A., Rodewald L.E., Humiston S.G., Raubertas R.F., Doane C.B., Szilagyi P.G. (1993). Accuracy of documented vaccination status of patients in pediatric emergency departments. Am. J. Dis. Child.

[16] Prendergast H.M., Graneto J., Kelley G.D. (2005). Child immunization status in an urban ED. Am. J. Emerg. Med..

[17] Vandenbroucke J.P., von Elm E., Altman D.G., Gøtzsche P.C., Mulrow C.D., Pocock S.J., Poole C., Schlesselman J.J., Egger M. (2014). Strengthening the Reporting of Observational Studies in Epidemiology (STROBE): Explanation and elaboration. Int. J. Surg..

[18] Australasian College for Emergency Medicine (2007). Emergency Triage Education Kit. https://acem.org.au/Content-Sources/Advancing-Emergency-Medicine/Better-Outcomes-for-Patients/Triage.

[19] Swiss Federal Office of Public Health (2018). Swiss Vaccination Plan 2018. Guidelines and Recommendations. https://www.bag.admin.ch/bag/de/home/gesund-leben/gesundheitsfoerderung-und-praevention/impfungen-prophylaxe/schweizerischer-impfplan.html.

[20] Swiss Federal Office for statistics (2014). Switzerland’s Areas with Urban Character 2012—A New Definition of Agglomerations and Other Urban Area Categories. https://www.bfs.admin.ch/bfsstatic/dam/assets/349566/master.

[21] Swiss Federal Office for Statistics (2020). International Definitions. https://www.bfs.admin.ch/bfs/de/home/statistiken/querschnittsthemen/raeumliche-analysen/raeumliche-gliederungen/internationale-definitionen.html.

[22] QGIS—A Free and Open Source Geographic Information System. https://qgis.org/en/site/.

[23] Geodesy and Federal Directorate of Cadastral Surveying and PLR-Cadastre Surveying Extracts from the Cadastre of Public-Law Restrictions on Landownership. https://www.cadastre.ch/en/services/service/extract.html.

[24] Alessandrini E.A., Alpern E.R., Chamberlain J.M., Shea J.A., Gorelick M.H. (2010). A new diagnosis grouping system for child emergency department visits. Acad. Emerg. Med..

[25] Bielicki J.A., Achermann R., Berger C. (2013). In touch but not up-to-date: Ambulatory visits and vaccination status in a cohort of young Swiss children. Vaccine.

[26] Schneider R., Reinau D., Schur N., Blozik E., Fruh M., Signorell A., Heininger U., Schwenkglenks M., Meier C.R. (2020). Coverage rates and timeliness of nationally recommended vaccinations in Swiss preschool children: A descriptive analysis using claims data. Vaccine.

[27] Weiss C., Schropfer D., Merten S. (2016). Parental attitudes towards measles vaccination in the canton of Aargau, Switzerland: A latent class analysis. BMC Infect. Dis..

[28] Stein-Zamir C., Israeli A. (2019). Timeliness and completeness of routine childhood vaccinations in young children residing in a district with recurrent vaccine-preventable disease outbreaks, Jerusalem, Israel. Eurosurveillance.

[29] Phadke V.K., Bednarczyk R.A., Salmon D.A., Omer S.B. (2016). Association between Vaccine Refusal and Vaccine-Preventable Diseases in the United States: A Review of Measles and Pertussis. JAMA.

[30] Salmon D.A., Dudley M.Z., Glanz J.M., Omer S.B. (2015). Vaccine hesitancy: Causes, consequences, and a call to action. Vaccine.

[31] Richard J.L., Masserey Spicher V. (2009). Large measles epidemic in Switzerland from 2006 to 2009: Consequences for the elimination of measles in Europe. Eurosurveillance.

[32] Siani A. (2019). Measles outbreaks in Italy: A paradigm of the re-emergence of vaccine-preventable diseases in developed countries. Prev. Med..

[33] MacDonald N.E. (2015). Vaccine hesitancy: Definition, scope and determinants. Vaccine.

[34] Simma L., Stocker M., Lehner M., Wehrli L., Righini-Grunder F. (2021). Critically Ill Children in a Swiss Pediatric Emergency Department with an Interdisciplinary Approach: A Prospective Cohort Study. Front. Pediatr..

[35] Krauss B.S., Harakal T., Fleisher G.R. (1991). The spectrum and frequency of illness presenting to a pediatric emergency department. Pediatr. Emerg. Care.

[36] Plumptre I., Tolppa T., Blair M. (2020). Parent and staff attitudes towards in-hospital opportunistic vaccination. Public Health.

[37] Baumer-Mouradian S.H., Kleinschmidt A., Servi A., Jaworski B., Lazarevic K., Kopetsky M., Nimmer M., Hanson T., Gray M.P., Drendel A.L. (2020). Vaccinating in the Emergency Department, a Novel Approach to Improve Influenza Vaccination Rates via a Quality Improvement Initiative. Pediatr. Qual. Saf..

[38] Hofstetter A.M., Schaffer S. (2021). Childhood and Adolescent Vaccination in Alternative Settings. Acad. Pediatr..

[39] Larson H.J., Clarke R.M., Jarrett C., Eckersberger E., Levine Z., Schulz W.S., Paterson P. (2018). Measuring trust in vaccination: A systematic review. Hum. Vaccin Immunother..

[40] Succi R.C.M. (2018). Vaccine refusal—What we need to know. J. Pediatr. (Rio. J.).

[41] Schiff J., Schmidt A.R., Pham P.K., Perez J.B., Pannaraj P.S., Chaudhari P.P., Liberman D.B. (2022). Parental attitudes in the pediatric emergency department about the COVID-19 vaccine. Vaccine.

[42] Schmid P., Rauber D., Betsch C., Lidolt G., Denker M.L. (2017). Barriers of Influenza Vaccination Intention and Behavior—A Systematic Review of Influenza Vaccine Hesitancy, 2005–2016. PLoS ONE.

[43] Sun X., Huang Z., Wagner A.L., Prosser L.A., Xu E., Ren J., Wang B., Yan W., Zikmund-Fisher B.J. (2018). The role of severity perceptions and beliefs in natural infections in Shanghai parents’ vaccine decision-making: A qualitative study. BMC Public Health.

[44] Cameron M.A., Bigos D., Festa C., Topol H., Rhee K.E. (2016). Missed Opportunity: Why Parents Refuse Influenza Vaccination for Their Hospitalized Children. Hosp. Pediatr..

